# The latent factor structure and assessment of childbirth-related PTSD: psychometric characteristics of the City Birth Trauma Scale—Persian version (City-BiTS-P)

**DOI:** 10.3389/fpsyt.2023.1204392

**Published:** 2023-06-20

**Authors:** Amin Vatanparast, Ali Kamrani, Shima Shakiba, Ramin Amouchie, Elnaz Akbari, Susan Ayers

**Affiliations:** ^1^Clinical Psychology, University of Social Welfare and Rehabilitation Sciences, Tehran, Iran; ^2^Occupational Therapy, University of Social Welfare and Rehabilitation Sciences, Tehran, Iran; ^3^Paramedical Department, Islamic Azad University, Rasht Branch, Rasht, Iran; ^4^Centre for Maternal and Child Health Research, School of Health Sciences, University of London, London, United Kingdom

**Keywords:** childbirth, PTSD, postpartum, Persian, factor structure

## Abstract

**Objective:**

There is no validated Persian questionnaire to measure childbirth-related PTSD (CB-PTSD) symptoms. To cover this gap, the present study aimed to provide a Persian version of the City Birth Trauma Scale (CityBiTS-Pr) and to determine its psychometric properties.

**Method:**

Since this is a cross-sectional study, sampling was done using a convenient sampling method. In total, 300 Persian-speaking women took part in this study and completed the City Birth Trauma Scale (CityBiTS-Pr), the Posttraumatic Stress Disorder Checklist for DSM-5 (PCL-5), the Edinburgh Postnatal Depression Scale (EPDS), the Anxiety Subscale of the Depression, and the Anxiety and Stress Scale (DASS-21). In addition, sociodemographic information was completed. A confirmatory factor analysis of two- and four-factor models and a bi-factor model with a general factor and two specific factors were tested. Fit indices were calculated for all three models. Reliability, convergent, divergent, and discriminant validity also were examined. R v4.2.1 and SPSS v23 were used for data analysis.

**Results:**

The four-factor model comprised intrusion, avoidance, negative cognitions and mood, and hyper-arousal showed a poor fit. The two-factor model composed of “birth-related symptoms” and “general symptoms” provided the best results based on all fit indices. The bi-factor result was relatively good, but the loadings indicated that the general symptoms factor is not well defined.

**Conclusion:**

The Persian version of the City Birth Trauma Scale (CityBiTS-Pr) is a valid and reliable questionnaire for evaluating postpartum PTSD.

## Introduction

Giving birth is one of the most important events a woman may experience. While for many women, childbirth is a positive and pleasant event, but sometimes, it can become traumatic (1, 2). In cases where childbirth is perceived as traumatic, the risk of post-traumatic stress disorder (PTSD) in the mother increases (3). Recent studies indicated that childbirth may cause childbirth-related PTSD (CB-PTSD) (2, 4–6), which is defined as a reaction to the experience of childbirth (7). Recent reviews and meta-analyses showed that CB-PTSD occurs in 3–4% of women in community samples (8–11), and this prevalence increases to a range of 15.7–19.5% in high-risk samples, including emergency cesarian section and severe pregnancy complications (10, 11).

The Diagnostic and Statistical Manual of Mental Disorders, fifth edition (DSM-5) (12), categorizes the symptoms of PTSD into four clusters as follows: re-experiencing aspects of the event, persistent avoidance of reminders of the event, negative cognitions and mood, and hyper-arousal, which should last more than 1 month to diagnose PTSD. Although childbirth is not an inherently traumatic event, for some women, childbirth generates a sense of threat of physical harm or death to themselves or their newborn, so the first criterion of PTSD (e.g., the existence of a traumatic stressor) is met (13, 14). In addition, unlike the other cases of PTSD, where people may avoid trauma-related reminders to reduce their anxiety, in CB-PTSD, the baby is an unavoidable reminder of the traumatic birth because they are constantly close to the mother and cannot be separated from her. This may cause strong negative feelings of blame and guilt in the mother (15).

Experiencing childbirth as a traumatic event plays an important role in developing CB-PTSD (16, 17). Vulnerability and risk factors include psychological trauma, especially a history of sexual abuse, emergency cesarean section, bleeding, stillbirth, loneliness, lack of support during childbirth, and so on, which are known as triggers of postpartum PTSD (18–22). Maternal CB-PTSD not only disrupts couples' relationships (23–25) but also has long-term negative effects on the infant's behavior and developmental process (26), all of which emphasize the importance of understanding, diagnosing, and minimizing the risk of CB-PTSD.

Most CB-PTSD studies have used measures of PTSD that have been developed for evaluating PTSD in other populations, such as veterans. Examples include the PTSD Symptom Self-Report (PSS-RS) (27) and the Impact of Event Scale (IES) (28). In addition to this, changes in the diagnostic criteria for PTSD in DSM-5 led to a need for questionnaire measures that specifically measured the symptoms of CB-PTSD according to these diagnostic criteria. To address this gap, Ayers et al. designed the City Birth Trauma Scale (CityBiTS) to assess CB-PTSD according to the DSM-5 criteria (29). In the original study, the questionnaire had high internal consistency and good psychometric properties (29). The results of exploratory factor analysis showed that the scale has a two-factor structure: the first factor, called “birth-related symptoms,” included symptoms of intrusion and avoidance and the second factor “general symptoms” consisted of negative cognition and mood and hyper-arousal symptoms (29).

Validation studies of the CityBiTS in other languages, including Hebrew, Croatian, French, Turkish, German, Brazilian, and Chinese, confirmed the two-factor structure of this scale (30–36). Moreover, in the Croatian and French studies (31, 32), the bi-factor model, including a general factor and two specific factors of the birth-related symptoms and general symptoms, showed a better fit than the two-factor structure. However, an extensive meta-analysis of the structure of PTSD symptoms in other populations showed that the two- and four-factor models of PTSD yielded a better model fit across studies (37).

To date, there is no validated Persian questionnaire to measure CB-PTSD. This is a serious hindrance in research and clinical settings. To cover this gap, the aim of the present study was to provide a Persian version of the City Birth Trauma Scale (CityBiTS-Pr) and to determine its psychometric properties.

## Materials and methods

### Procedure and participants

The study design is a cross-sectional study. The population of the present study included all mothers who gave birth in several hospitals in Tehran between April and August 2022. The inclusion criteria were women who were able to understand and speak Persian and women who were in the 1^st^ year of the postpartum period. The study procedure was approved by the ethics committee of the University of Social Welfare and Rehabilitation Sciences (Reg. IR.USWR.REC.1401.143). Data were collected through the self-report method in the private interview room allocated in the hospital. In the first step, all mothers signed a version of the informed consent form prior to participating in the study. The sociodemographic information was collected in an interview format by research assistants. All other information was gathered with pen and paper, with research assistants being available for any possible questions and explanations. For evaluating test–re-test consistency over time, the women who participated in the first assessment were re-invited to the hospitals 2 weeks later. In the present study, 30 people returned and completed the questionnaires at that time.

According to the recommended parameters for factor analysis studies (38), a total number of 290 participants were needed for the present study. To cover the possible withdrawal, the number of participants was increased to 325. Twenty-five participants left the questionnaires incomplete, so the final sample size was 300 women.

### Measures

#### Sociodemographic and medical data

The sociodemographic sheet contained questions about the mother's age, marital status, educational level, financial status, history of a traumatic event, history of traumatic childbirth, parity, and mood of delivery.

#### City Birth Trauma Scale

The CityBiTS is a 29-item self-report questionnaire developed to assess birth-related symptoms on a Likert-type scale ranging from 0 (not at all) to 3 (5 or more times) according to the DSM-5 criteria (29). The CityBiTS measures whether the birth complications met the criteria for a traumatic stressor (items 1–2) and symptoms of re-experiencing (items 3–7), avoidance (items 8–9), negative cognitions and mood (items 10–16), and hyper-arousal (items 17–22). Furthermore, items 23 and 24 are associated with depersonalization/derealization symptoms (a dissociative subtype of PTSD). The scores pertaining to items 3–22 (the main 20 items of the questionnaire that should be considered in scoring) range from 0 to 60. Higher scores indicate more severe symptoms of PTSD. Four additional items evaluate the onset of PTSD symptoms (item 25: 0 = prior to birth and 2 = delayed onset), duration (item 26), distress and impairment (items 27 and 28), and the last item (29) pertains to exclusion criteria. The CityBiTS contains two subscales of birth-related symptoms and general symptoms. The birth-related symptoms comprise symptoms of intrusion and avoidance and two items of negative cognitions and mood. In contrast, the general symptoms include two other symptoms of negative cognition and mood and hyper-arousal. In the original study, Cronbach's alpha for the symptom subscales and total subscales were 0.83–0.88 and 0.92, respectively (29). After obtaining permission from the developer of the questionnaire (Susan Ayers), the CityBiTS was translated into Persian using the back-translation method. The back-translated version then was discussed with Susan Ayers, which led to minor revisions in wording.

#### Post-traumatic stress disorder checklist for DSM-5 (PCL-5)

The PCL-5 measures the frequency and severity of PTSD symptoms during the last month (39). This 20-item self-report questionnaire covers PTSD symptoms in four clusters: intrusion (items 1–5), avoidance (items 7–6), negative cognitions and mood (items 8–14), and hyper-arousal (items 15–20). The range of response options is from 0 (not at all) to 4 (extremely), and the total score is obtained from the sum of the items' scores, and higher scores indicate greater severity of PTSD symptoms. The PCL has been shown to have very good internal consistency (alpha = 0.94) and test re-test reliability (re-test r = 0.88, 1-week interval), and it correlates strongly (i.e., r > 0.75) with other instruments of PTSD symptomatology (40). The Persian version of this scale also showed good psychometric properties (41).

#### Edinburgh postnatal depression scale

The EPDS is a 10-item self-report questionnaire that assesses the symptoms of postpartum depression on a four-point Likert scale from 0 to 3 (42). The total scores range from 0 to 30, and higher scores reflect greater levels of depression. A cutoff of ≥10 shows significant levels of depression, indicating that a more effective diagnostic procedure should be carried out. Good psychometric properties have been reported for the Persian version of this questionnaire (43).

#### Anxiety subscale of the depression, anxiety, and stress scale

The Depression, Anxiety, and Stress Scale-21 (DASS-21) is a 21-item self-report questionnaire that measures the severity and frequency of the three negative states of depression, anxiety, and stress during the past week (44). The DASS-21 is a four-point Likert scale, and each emotional state is measured with seven items. The internal consistency of the Persian version of this scale for three emotional states of depression, anxiety, and, stress was 0.85, 0.85, and 0.87, respectively (45). In the present study, only the anxiety subscale was used.

### Statistical analysis

For data analysis, the range of the CityBiTS-Pr questionnaire items was checked, and then, the confirmatory factor analysis of two- and four-related factors was tested with the WLSMV estimator. After that, a bi-factor model with a higher order general factor as well as the two specific factors was checked by the WLSMV estimator, and then, the fit indices were calculated for all three models.

The reliability of the questionnaire by calculating internal consistency with Cronbach's alpha and test–re-test using intraclass correlation by a two-way fixed model and average measure with an absolute agreement was examined. The convergent and divergent validity of the questionnaire was checked through Spearman correlation with other questionnaires. The differential validity of the questionnaire for the study groups was performed using Student's *t*-test and analysis of variance. In places where it was not possible to use the analysis of variance due to data distribution or heterogeneity of variance between groups, the Kruskal–Wallis test was used. Bonferroni correction was used for comparisons in *post-hoc* tests.

The confirmatory factor analysis and bi-factor model were examined by R v4.2.1 under Rstudio 2022.02.3 software and using “*lavaan*” and “*Bi-factorIndicesCalculator*” packages. All other statistical analyses were performed by SPSS v23 Windows Edition. A significance level of 0.05 was used in all tests.

## Results

The descriptive analysis of the data showed that participants used the full range of answers for all the questions of the CityBiTS except for questions no. 21 and 22. The mean age of the participant was 29.91 (SD 4.58), and nearly all participants were married. Other descriptive statistics of the sample are shown in Table 1.

**Table 1:** Descriptive characteristics of the participants.

**Variable**	**Level**	**n**	**%**
Education	Diploma	97	32.3
	Associate	60	20
	Bachelor	111	37
	Master	31	10.3
Marital status	Single	1	0.3
	Married	294	98
	Widow	1	0.3
Socioeconomic	Lower class	56	18.7
	Middle class	169	56.3
	Upper class	71	23.7
History of trauma/abuse	No	240	80
	Yes	59	19.7
History of traumatic birth	No	179	59.7
	Yes	116	38.7
Type of birth	Natural	78	26
	Requested cesarean	153	51
	Emergency cesarean	67	22.3
Number of births	No	192	64
	Just one	81	27
	Two or more	26	8.7

### Confirmatory factor analysis

According to the results of previous studies, the confirmatory factor analysis was tested based on the three different models. First, the four-factor model of intrusions, avoidance, negative cognitions and mood, and hyper-arousal was tested. This analysis showed a poor fit, X^2^(164) = 688.095, X^2^/df = 4.19, CFI = 0.628, TLI = 0.569, and RMSEA = 0.103 ci90 (0.095, 0.111). Second, the two-factor model composed of “birth-related symptoms” and “general symptoms” in accordance with the original validation was tested. The results of this analysis provided better results based on all fit indices, X^2^(169) = 245.630, X^2^/df = 1.453, CFI = 0.946, TLI = 0.939, RMSEA = 0.039 ci90 (0.028, 0.049). The loadings for the two-factor confirmatory factor analysis are shown in Table 2.

**Table 2:** Standardized factor loadings for the two-factor model of the CityBiTS-P (*n* = 300).

**Items**	**Factor loadings**	
	**BRS**	**GS**
3. Recurrent unwanted memories of the birth…	0.877	
4. Bad dreams or nightmares about the birth …	0.819	
5. Flashbacks to the birth and/or reliving the experience.	0.824	
6. Getting upset when reminded of the birth.	0.871	
7. Feeling tense or anxious when reminded of the birth.	0.813	
8. Trying to avoid thinking about the birth.	0.806	
9. Trying to avoid things that remind me of the birth …	0.830	
10. Not able to remember details of the birth.	0.766	
11. Blaming myself or others for what happened during the birth.	0.873	
12. Feeling strong negative emotions about the birth.	0.796	
13. Feeling negative about myself or thinking something awful will happen.		0.733
14. Lost interest in activities that were important to me.		0.742
15. Feeling detached from other people…		0.731
16. Not able to feel positive emotions …		0.766
17. Feeling irritable or aggressive.		0.789
18. Feeling self-destructive or acting recklessly.		0.753
19. Feeling tense and on edge.		0.805
20. Feeling jumpy or easily startled.		0.813
21. Problems concentrating.		0.795
22. Not sleeping well … not due to the baby's sleep pattern.		0.750

Finally, we tested the bi-factor model with a general factor as well as the birth-related symptoms and general symptom, which revealed a relatively good fit for the data, χ^2^ (150) = 246.909, χ^2^/df = 1.64, CFI = 0.931, TLI = 0.913, and RMSEA = 0.046 ci90 (0.036, 0.057). The ECV indices showed that 56% of the common variance across the items is related to the general factor. The hierarchical omega of the general factor was ωH = 0.70, which is below the 0.80 cutoff value. Items 13, 19, 20, 21, and 22 were not significantly loaded on the general symptoms factor, and the IECV for these items was higher than 0.95, which indicates that these items reflect the general factor. Comparing the three mentioned models, their fit indices and pattern of loadings revealed that the best model for this questionnaire is the two-factor model. As mentioned earlier, the bi-factor results were relatively good, but the loading findings indicated that the general symptoms factor is not well defined.

### Reliability

The analysis of the internal consistency by using Cronbach's alpha showed very high reliability for the entire questionnaire (alpha = 0.949). Cronbach's alpha for the birth-related symptoms and the general symptoms was 0.955 and 0.937, respectively. Inter-item correlations were between 0.49 and 0.81 for the birth-related symptoms and 0.49 and 0.80 for the general symptoms. The correlation between the items for the whole questionnaire ranged from 0.27 to 0.81.

The test–re-test reliability of the questionnaire was assessed *via* ICC with a two-way fixed model, with an average measure with an absolute agreement. The total score of the CityBiTS-Pr had a reliability of 0.967 with 95% confidence intervals ranging from 0.93 to 0.98. The reliability of the birth-related symptoms and the general symptoms was 0.966 and 0.949, respectively.

### Convergent validity and divergent validity

The convergent validity of the CityBiTS-Pr and its subscales was tested through the correlation with the total score of the PCL-5 total score (Table 3). The results showed a high and significant correlation between the PCL-5 total score and the total score (r = 0.805^**^) and both subscales of the CityBiTS-Pr. The correlation of the PCL-5 with the birth-related symptoms (r = 0.724^**^) was higher than the general symptoms (r = 0.674^**^).

**Table 3:** Pearson's correlations of the CityBiTS-Pr and subscales, with DASS-A, EPDS, and PCL-5 (*n* = 300).

	**1**	**2**	**3**	**4**	**5**	**6**
1-CityBiTS total score	-					
2-Birth-related symptoms	0.913^**^	-				
3-General symptoms	0.817^**^	0.511^**^	-			
4-EPDS	0.387^**^	0.299^**^	0.394^**^	-		
5-DASS-A	0.442^**^	0.379^**^	0.396^**^	0.418^**^	-	
6-PCL-5	0.805^**^	0.724^**^	0.674^**^	0.397^**^	0.509^**^	-

^**^p-value < 0.01.

The divergent validity of the questionnaire was investigated *via* the correlation of the CityBiTS-Pr with the Edinburgh Postnatal Depression Scale (EPDS) and the DASS anxiety subscale (Table 3). The results revealed that the CityBiTS-Pr total score (r = 0.387^**^) as well as both birth-related symptoms (r = 0.299^**^) and general symptoms (r = 0.394^**^) had a significant correlation with depression. The correlation of anxiety with the CityBiTS-Pr total score and its subscales was also significant.

### Discriminant validity

The discriminant validity of the questionnaire was evaluated by examining the total score of the questionnaire and subscales in the studied groups (Table 4). Women who experienced an emergency birth reported significantly higher scores in the total score and both subscales (general and birth-related symptoms) compared to women who had a natural birth or planned cesarean delivery. Those with a history of traumatic events showed significantly higher scores in the total score (t = 8.69, df = 297, *p*-value < 0.001) as well as in both subscales (BRS: t = 8.74, df = 297, *p*-value < 0.001; GS: t = 4.76, df = 75.50, *p*-value < 0.001). Evaluating the effect of traumatic birth showed that people who evaluated their birth as a traumatic event had significantly higher scores in the total score (t = 11.37, df = 192.4, *p*-value < 0.001) and both subscales (BRS: t = 13.00, df = 201.96, *p*-value < 0.001; GS: t = 5.68, df = 191.20, *p*-value < 0.001) than those who did not evaluate their birth as traumatic. Considering that the majority of the participants were married people, the effect of the type of marriage could not be investigated.

**Table 4:** Differences in the CityBiTS-Pr and subscales between known groups.

		**n**	**Birth-related symptoms**		**General symptoms**		**Total score**	
**Variables**	**Groups**		***M***	***SD***	***M***	***SD***	***M***	***SD***
Type of birth	Natural birth	78	4.94	6.5	3.35	4.864	8.28	10.2
	Planned cesarean	153	7.18	6.0	3.61	4.543	10.78	8.8
	Emergency cesarean	67	14.52	6.1	6.63	5.559	21.15	10.1
			(BRS: F = 47.2, df = 2/295, *p*-value < 0.001)		(GS: F = 10.64, df = 2/295, *p*-value < 0.001)		(F = 37.643, df = 2/295, *p*-value < 0.001)	
History of traumatic birth	No	179	4.62	4.947	2.91	4.073	7.53	7.446
	Yes	116	13.72	6.391	6.36	5.661	20.08	10.259
			(BRS: t = 13.00, df = 201.96, *p*-value < 0.001)		(GS: t = 5.68, df = 191.20, *p*-value < 0.001)		(t = 11.37, df = 192.4, *p*-value < 0.001)	
History of trauma/abuse	No	240	6.57	6.339	3.45	4.486	10.01	9.402
	Yes	59	14.63	6.370	7.34	5.865	21.97	9.699
			(BRS: t = 8.74, df = 297, *p*-value < 0.001)		(GS: t = 4.76, df = 75.50, *p*-value < 0.001)		(t = 8.69, df = 297, *p*-value < 0.001)	

## Discussion

This study was conducted to evaluate the psychometric properties of the Persian version of the City Birth Trauma Scale (CityBiTS-Pr) in a convenience sample of women, who gave birth in the past year. The original version of the CityBiTS was recently developed to measure CB-PTSD symptoms among English-speaking women (29). To date, the validity of the CityBiTS has been examined in seven countries (30–36). Consistent with previous versions, the Persian version of the CityBiTS (CityBiTS-Pr) was found to be a reliable and valid instrument.

In the present study, the reliability of the CityBiTS-Pr was examined via internal consistency, as well as test–re-test analyses, which in line with previous validation studies, the CityBiTS-Pr was found to be a reliable questionnaire. Cronbach's alpha for the total scale and both subscales was high at 0.9, which indicates very good internal consistency. Regarding test–re-test reliability, the significant correlation of the CityBiTS-Pr over a 2-week period showed that the scores over time were stable.

According to the DSM-5 (12), PTSD is defined as a disorder composed of a four-cluster structure (i.e., intrusion/re-experience, avoidance, negative cognitions and mood, and hyper-arousal). However, as the four-factor model showed a poor fit to data, the four-cluster model was not supported by the current study. This finding is in agreement with other birth-related PTSD findings (29, 30, 34, 46). Consistent with the Hebrew and original validation studies of the CityBiTS (29, 30), the results of the two-factor solution with two correlated dimensions of the birth-related symptoms and the general symptoms provided the best fit. This finding is not in line with those reported by PTSD scales used in other populations, where the presence of several factors is common (47–49). The bi-factor model CFA also revealed a relatively good fit. The ECV showed that 56% of the common variance across the items was related to the general symptoms factor, but the loadings indicated that the general symptoms factor is not well defined. From all items of the questionnaire, the items 13, 19, 20, 21, and 22 did not significantly load on the general symptoms factor. These findings are not congruent with the French and Croatian validation studies (31, 32) where, similar to the present study, in addition to the four and two clusters, the bi-factor models also were examined, but their findings showed that the bi-factor model was the best-fitted model.

The convergent and divergent validity were examined using the PCL-5, the EPDS, and the anxiety subscale of the DASS-21 instruments. Our results revealed high convergent validity for both the birth-related symptoms and the general symptoms of the CityBiTS-Pr, with its significant correlation with the PTSD total score of the PCL-5, indicating that both factors are related to PTSD symptoms. More specifically, in accordance with the previous studies (31, 32), birth-related symptoms showed greater correlations with the measures of PTSD. Depression and anxiety scores were used to evaluate the divergent validity of the CityBiTS-Pr. The good divergent validity of our study was in line with the prior findings, which means that, similar to the earlier studies, which reported a significant correlation between PTSD and depression/anxiety scores (31, 32, 34), we also found a moderate correlation. Therefore, the divergent validity could also be confirmed.

The known groups' method was used to evaluate discriminant validity. The total scores of the CityBiTS-Pr and its subscales were significantly higher in participants who evaluated their childbirth as a traumatic event or had a history of abuse in their own lives. This finding is in line with the prior literature (50–52) that a history of abuse, regardless of whether it is physical, sexual, or emotional, increases the scores of the CityBiTS. Women with a history of trauma are more vulnerable to traumatic events. Therefore, trauma scores in this group were expected to be higher than women who had not experienced such events. According to the literature's findings (53–55), an emergency cesarean is associated with increased CityBiTS scores. Similarly, in the current study, women who had an emergency cesarean reported higher scores on the total scale of the CityBiTS-Pr and both birth-related and general symptoms subscale compared to women who had natural births or planned cesareans. While, in the studies of Nakić Radoš et al. and Sandoz et al. (31, 32), the emergency cesarean raised the scores of the total scales and just birth-related symptoms. It should be also mentioned that, as expected, the rate of cesarean section in the current study similar to many other developing countries was considerably high (73%). According to the studies, the main reason for requesting cesareans in Iran is the fear of labor birth (56, 57). However, there are also some other factors that affect the excessively increasing rate of cesarean in Iran such as people's education, career, age, and place of living (58).

This study also has some limitations. First, almost all participants (98%) of the present study were in a couple's relationship, and more than half of them (67/3%) had a university degree. Therefore, findings cannot be generalized to the general population of Iran, where the proportion of women without a partner and lower education levels is higher than the current sample. Second, no data about ethnical diversity were collected, and thus, further research is needed to examine the psychometric properties of the CityBiTS in different ethnic populations. Finally, an important limitation of this study is using a cross-sectional design, which is a major hindrance to providing information about the trajectory of symptoms over time. In this regard, future studies should use longitudinal studies and structured clinical interviews in addition to self-report instruments to examine the trajectory of postpartum symptoms.

In conclusion, the Persian version of the City Birth Trauma Scale (CityBiTS-Pr) is a reliable and valid questionnaire, which can be used to measure postpartum PTSD symptoms according to the DSM-5 criteria. It demonstrated very high internal consistency for the total scale and both subscales, as well as consistent measures over time. Two factors of birth-related symptoms and general symptoms were identified. Furthermore, the Persian version of the CityBiTS (CityBiTS-Pr) is an applicable instrument for both research and clinical practice.

## Data availability statement

The raw data supporting the conclusions of this article will be made available by the authors, without undue reservation.

## Ethics statement

The studies involving human participants were reviewed and approved by Ethics Committee of the University of Social Welfare and Rehabilitation Sciences. The patients/participants provided their written informed consent to participate in this study.

## Author contributions

AV, SS, SA, and AK contributed to conception and design of the study. EA and RA gathered the data. AK performed the statistical analysis. AV and AK wrote the first draft of the manuscript. SS, SA, EA, and RA wrote sections of the manuscript. All authors contributed to manuscript revision, read, and approved the submitted version.
